# Synergistic therapy for osteoporosis: a soybean osteogenic peptide-loaded fish gelatin/κ-carrageenan gel for enhanced bone regeneration

**DOI:** 10.3389/fnut.2025.1735717

**Published:** 2025-12-18

**Authors:** Jinpeng Gong, Yuhan Zhang, Tao Jie, Tao Huang, Yupeng Ma, Hao Chen, Jing Gan, Junbo Ge

**Affiliations:** 1 Department of Trauma Orthopedics, Yantaishan Hospital Affiliated to Binzhou Medical University, Yantai, China; 2 Marine College, Shandong University, Weihai, Shandong, China; 3 College of Life Science, Yantai University, Yantai, Shandong, China

**Keywords:** dual-network gel, fish gelatin, κ-carrageenan, osteoporosis, soybean osteogenic peptide

## Abstract

Osteoporosis is a widespread skeletal disorder associated with reduced bone formation and increased fracture risk. Peptide-based therapeutics offer demonstrate anabolic potential for osteoporosis management but are limited by instability and rapid clearance. In this study, a dual-network hydrogel composed of fish gelatin (FG) and κ-carrageenan (κ-CG) was developed as a biocompatible carrier for a soybean-derived osteogenic peptide (SOP). The FG/κ-CG system exhibited provided mechanical integrity, hydration balance, and thermal stability, thereby enabling sustained peptide protection and controlled release. In a glucocorticoid-induced zebrafish osteoporosis model, oral administration of the SOP-loaded gel effectively restored bone mineralization to a level comparable with alendronate treatment. These findings suggest that the FG/κ-CG-SOP hydrogel provides a stable and bioactive platform for osteogenic peptide delivery and represents a promising nutritional or therapeutic approach for osteoporosis management.

## Graphical abstract

**Figure:**
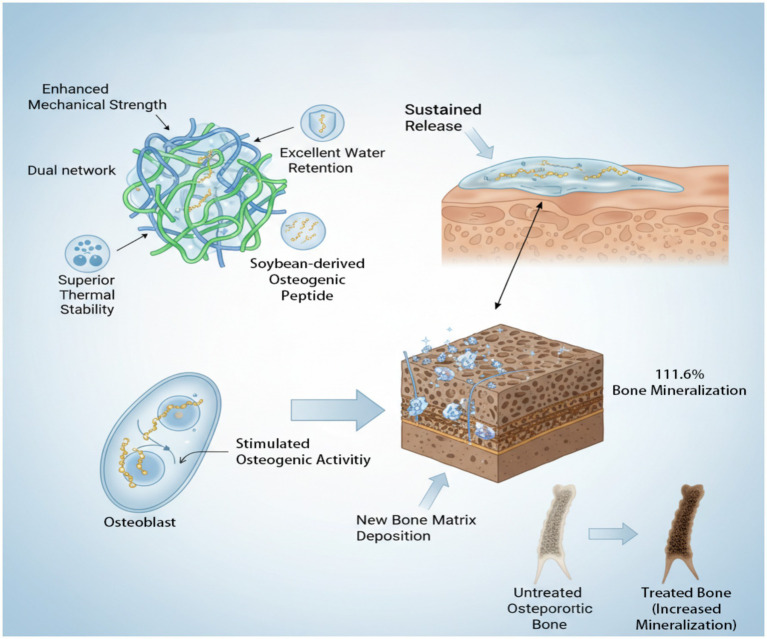

## 1 Introduction

Osteoporosis is a systemic skeletal disorder characterized by decreased bone mass, structural deterioration, and increased fracture susceptibility (1–3). Its pathogenesis mainly arises results from an imbalance between bone resorption and formation (4). Current therapeutic strategies, including bisphosphonates, hormone therapy, and monoclonal antibodies, can slow bone loss to some degree; however, their long-term efficacy is limited undermined by adverse side effects and considerable variability in patient response (5–7). These limitations highlight the urgent need for novel therapeutic approaches that directly restore osteogenic capacity within osteoporotic bone tissue.

Bioactive peptides have recently attracted gained increasing attention as emerging osteoinductive agents (8, 9). Compared with conventional small-molecule drugs and protein growth factors, peptides offer possess well-defined molecular weights, high aqueous solubility, low immunogenicity, and the ability to mimic functional motifs of natural proteins (10–12). These properties enable them to regulate key osteoblast activities, including adhesion, proliferation, differentiation, and mineralization. Among them, the soybean-derived osteogenic peptide (SOP, amino acid sequence: VVELLKAFEEKF) has demonstrated notable osteogenic potential in preclinical studies. By activating the TGF-β1/Smad and p38 MAPK signaling pathways, SOP promotes MC3T3-E1 pre-osteoblast proliferation and differentiation, thereby counteracting the osteogenic suppression characteristic of osteoporotic microenvironments. It also enhances the expression of osteogenic markers such as alkaline phosphatase (ALP) and osteocalcin (OCN) (13, 14). Despite these advantages, SOP, like most hydrophilic peptides, is prone to undergoes enzymatic degradation, rapid systemic clearance, and inefficient targeted delivery, which restricts its therapeutic utility *in vivo* (15, 16).

To address these limitations, the development of protective and sustained-release carriers is critical. Natural polymer-based gels have emerged as promising delivery systems due to their high water content, excellent biocompatibility, and three-dimensional network structures that resemble the extracellular matrix (ECM) (17–19). For instance, FG, a collagen-derived protein obtained from fish skin, bones, and scales, supports osteoblast adhesion and exhibits tunable degradation properties, making it a suitable carrier for peptides (20). However, FG gels suffer from poor thermal stability, with melting points below 40 °C, which limits their use in sterilization and long-term implantation (21, 22). Conversely, κ-CG, a sulfated polysaccharide derived from red algae, forms mechanically robust and biocompatible gels with excellent gelation properties. Yet, κ-CG gel alone is brittle and prone to dehydration-induced shrinkage, which compromises its structural stability.

By combining the complementary properties of FG and κ-CG, a dual-network gel can be engineered to provide both enhanced mechanical strength and favorable biological functionality (23, 24). In this study, we developed a 6.8% FG + 1.2% κ-CG dual-network gel loaded with SOP, systematically characterized its physicochemical properties, and evaluated its therapeutic efficacy in a glucocorticoid-induced osteoporotic zebrafish model. Our findings demonstrate indicate that this functionalized gel effectively restores bone formation under osteoporotic conditions, offering a promising platform for peptide-based osteoporosis therapy.

## 2 Results and discussion

### 2.1 Appearance and color characteristics

**Figure 1:**
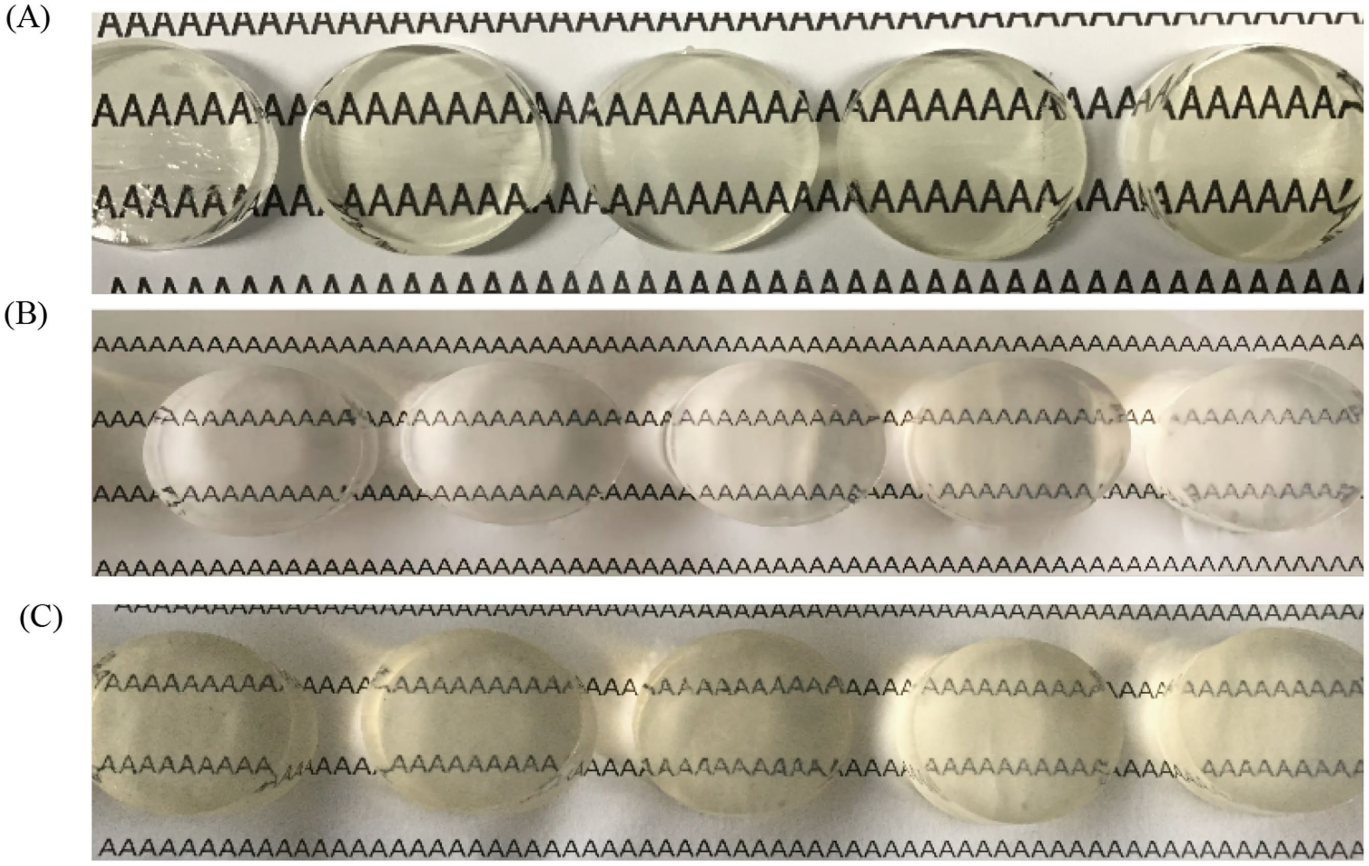
Visual appearance of gels. **(A)** Visual appearance of FG gel as concentration increases [from left to right: 6% (w/v), 7% (w/v), 8% (w/v), 9% (w/v), 10% (w/v)]. **(B)** Visual appearance of κ-CG gel with increasing concentration [from left to right: 0.8% (w/v), 1.0% (w/v), 1.2% (w/v), 1.4% (w/v), 1.6% (w/v)]. **(C)** Visual appearance of composite gels with increasing concentration [from left to right: 7.2% (w/v) FG + 0.8% (w/v) κ-CG–6.4% (w/v) FG + 1.6% (w/v) κ-CG].

Sensory evaluation and colorimetric analysis provided complementary perspectives on the visual properties of gels. FG gel (6–10% w/v) exhibited increasing strength and hardness with rising concentration, mainly due to hydrogen bonding and van der Waals interactions facilitating triple-helix reorganization and forming a compact physical network. However, transparency gradually decreased with concentration because of reduced pore size and enhanced light scattering. At high concentrations, FG gel showed mild syneresis and a yellowish hue (Figure 1A).

By contrast, κ-CG gel (0.8–1.6% w/v) exhibited much greater hardness at equivalent concentrations. This superior mechanical strength was attributed to ionic bridging between sulfate groups and K^+^ ions that stabilized the rigid double-helix bundles. Nevertheless, their rigidity also resulted in greater brittleness and more pronounced syneresis, which diminished with increasing concentration. Initially, κ-CG gel remained colorless and transparent (Figure 1B).

Composite FG/κ-CG dual-network gels combined the two structural features. Introduction of κ-CG significantly increased strength and hardness through synergistic interpenetration of FG triple helices and κ-CG double helices. With increasing κ-CG content, transparency decreased due to network densification and light scattering, while samples became milky white. Brittleness decreased with higher κ-CG but remained higher than that of the FG gel alone. This balanced regulation confirmed that the dual network structure not only enhanced strength but also influenced optical appearance (Figure 1C).

**Figure 2:**
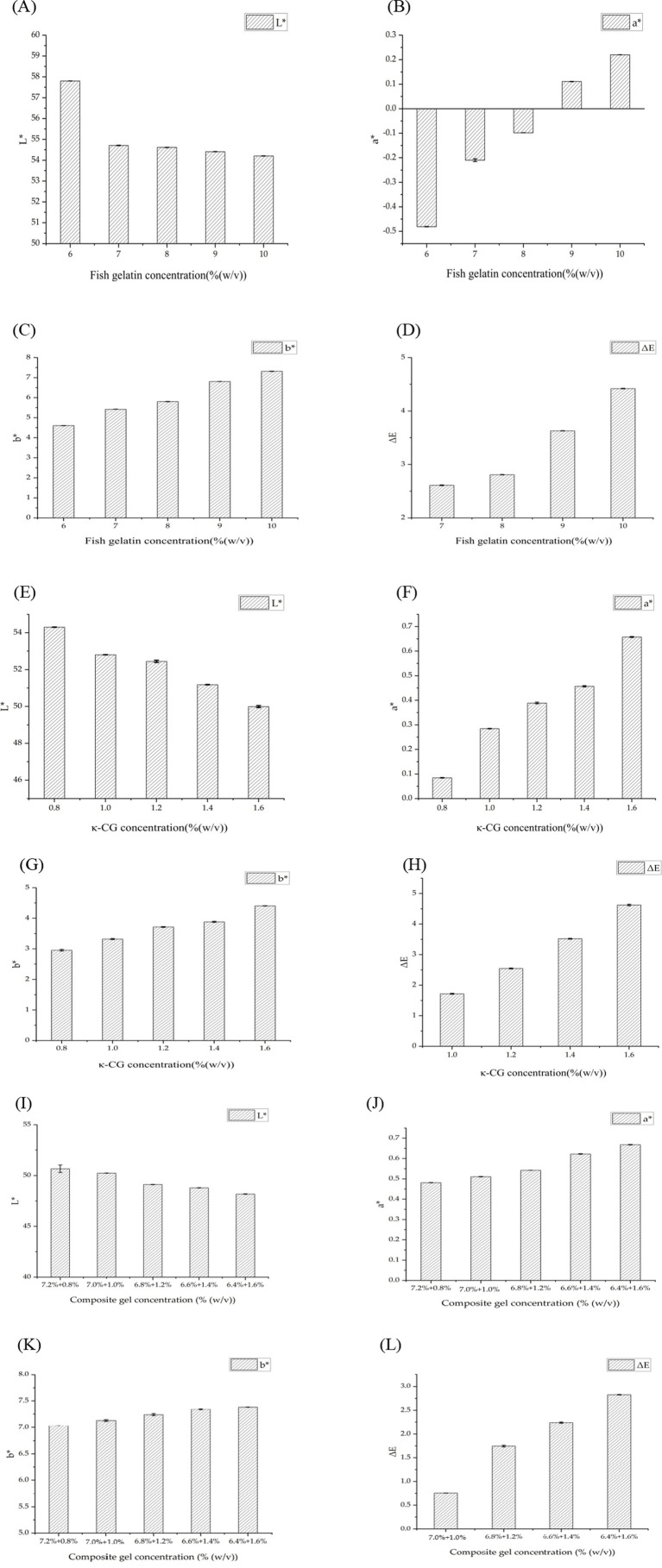
Comparison of color characteristics: **(A)** L* value (lightness) of FG gel; **(B)** a* value (red-green component) of FG gel; **(C)** b* value (yellow-blue component) of FG gel; **(D)** ΔE (total color difference) of FG gel compared to 6% (w/v) FG gel; **(E)** L* value (lightness) of κ-CG gel; **(F)** a* value (red-green component) of κ-CG gel; **(G)** b* value (yellow-blue component) of κ-CG gel; **(H)** ΔE (total color difference) of κ-CG gel compared to 0.8% (w/v) κ-CG gel; **(I)** L* values (lightness) of composite FG/κ-CG gels; **(J)** a* values (red-green component) of composite FG/κ-CG gels; **(K)** b* values (yellow-blue component) of composite FG/κ-CG gels; **(L)** ΔE (total color difference) of composite FG/κ-CG gels. Mean ± SD, *n* = 3.

Colorimetric analysis corroborated these findings. For FG gel, increasing concentration reduced lightness (L*) while elevating redness (a*) and yellowness (b*) (Figures 2A–C). κ-CG gel showed a similar concentration-dependent pattern, although their overall L* values were lower than those of FG gel, likely due to the intrinsic optical properties of polysaccharides (Figures 2E–G). Composite FG/κ-CG gels also exhibited concentration-dependent color changes: higher κ-CG content led to decreased L* and increased a* and b* values (Figures 2I–K), consistent with the visual observation of reduced transparency and a shift toward a milky-white appearance. Despite these variations, the total color difference (ΔE) values for all three systems remained below 3.2 (Figures 2D,H,L), a threshold generally considered imperceptible to the human eye. Overall, although transparency was compromised with increasing concentration, especially in composite gels, the gels maintained acceptable visual uniformity while achieving enhanced structural robustness.

The color difference (ΔE) among different formulations reflects the uniformity and crosslinking density of the polymeric network. These structural variations are directly related to peptide diffusion and retention within the gel matrix, thus influencing the subsequent hydration and release behavior. Therefore, the observed color variation provides an early indication of network compactness, which in turn affects biological performance such as nutrient diffusion and cell interaction.

### 2.2 Water content

**Figure 3:**
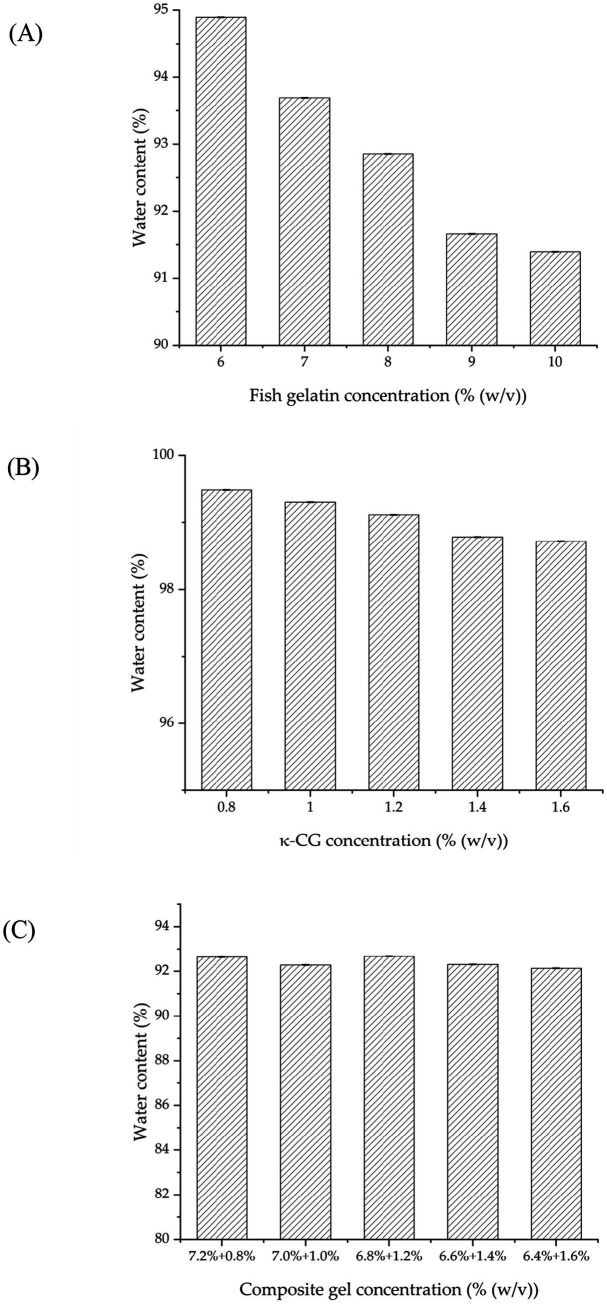
Equilibrium water content of **(A)** FG gel, **(B)** κ-CG gel, and **(C)** FG/κ-CG composite gels. Mean ± SD, *n* = 3.

Equilibrium water content (WC) of FG gel and κ-CG gel single networks declined with increasing concentration because denser molecular packing reduced free volume and more crosslinking points restricted chain extension (Figures 3A,B). Similar decreases were seen in composite gels, which showed slightly lower WC than FG gel alone at equivalent polymer content, due to network densification (Figure 3C). Nevertheless, all gels maintained WC above 90%. Such high hydration supports moist microenvironments, nutrient exchange, cellular metabolism, and osteoblast migration, providing favorable conditions for bone regeneration (25).

### 2.3 Rehydration rates

**Figure 4:**
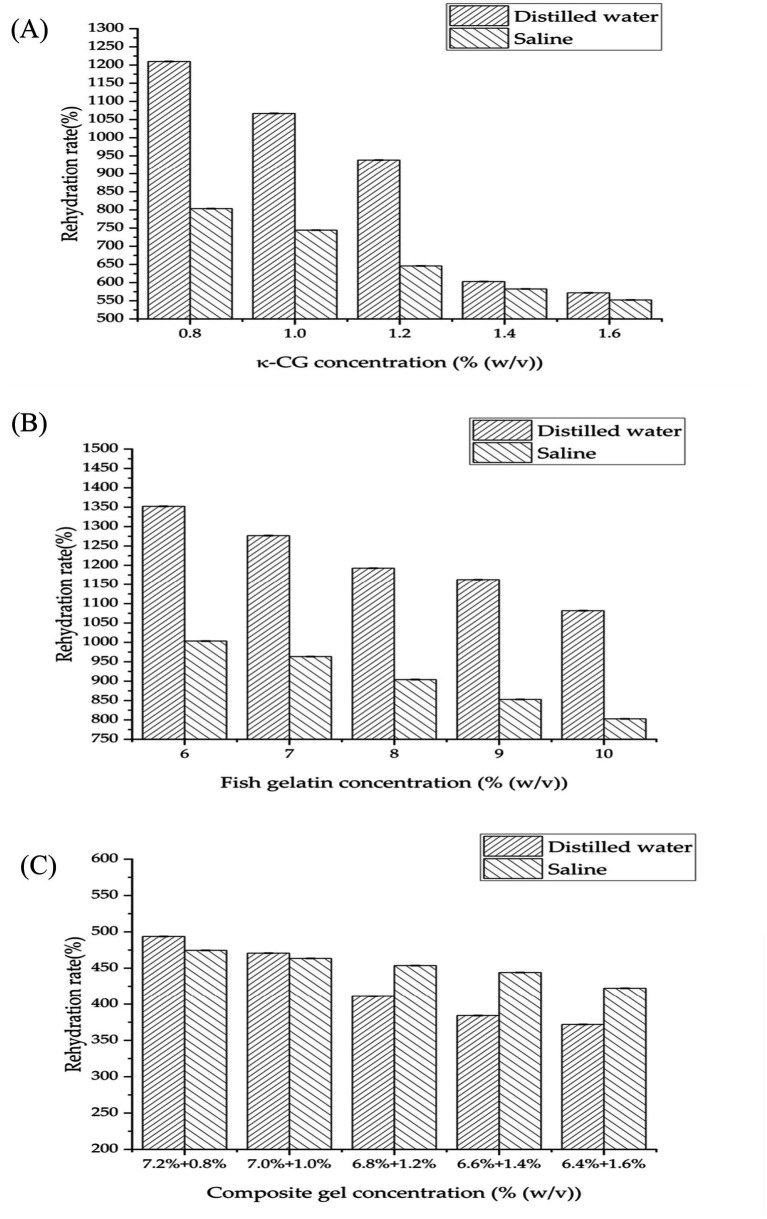
Rehydration rates of gels in distilled water and saline (0.9% NaCl solution) at varying concentrations. **(A)** κ-CG gel; **(B)** FG gel; **(C)** FG/κ-CG composite gels. Mean ± SD, *n* = 3.

Rehydration rates varied significantly across systems. κ-CG gel showed high uptake (1,200 and 800%), but demonstrated strong ionic sensitivity due to Na^+^ shielding of sulfate ester charges (Figure 4A) (26). FG gel exhibited exceptionally high rehydration capacity, peaking at 1,350% in distilled water and 1,000% in saline (Figure 4B). In both systems, increasing concentration reduced rehydration, caused by denser networks limiting water penetration.

FG/κ-CG composite gels exhibited dramatically lower rehydration (max 494% in water, 475% in saline), as the interpenetrating network physically restricted swelling (Figure 4C). The limited expansibility of this hydrogel makes it particularly suitable for osteoporotic environments. It can maintain its own mechanical properties without exerting destructive stress on the already damaged bone tissue (27). Thus, the dual network prevents over-swelling while retaining hydration, highlighting its suitability for *in vivo* applications.

Hydration behavior is a critical factor for biocompatibility and nutrient exchange in vivo. The high water content of the FG/κ-CG gel ensures sufficient hydration for peptide diffusion, while its controlled rehydration prevents excessive swelling that could weaken the matrix or cause mechanical mismatch with surrounding tissue. This balance between water uptake and structural stability supports offers a favorable environment for peptide preservation and sustained bioactivity.

### 2.4 Rheological properties

#### 2.4.1 Frequency-dependent behavior

**Figure 5:**
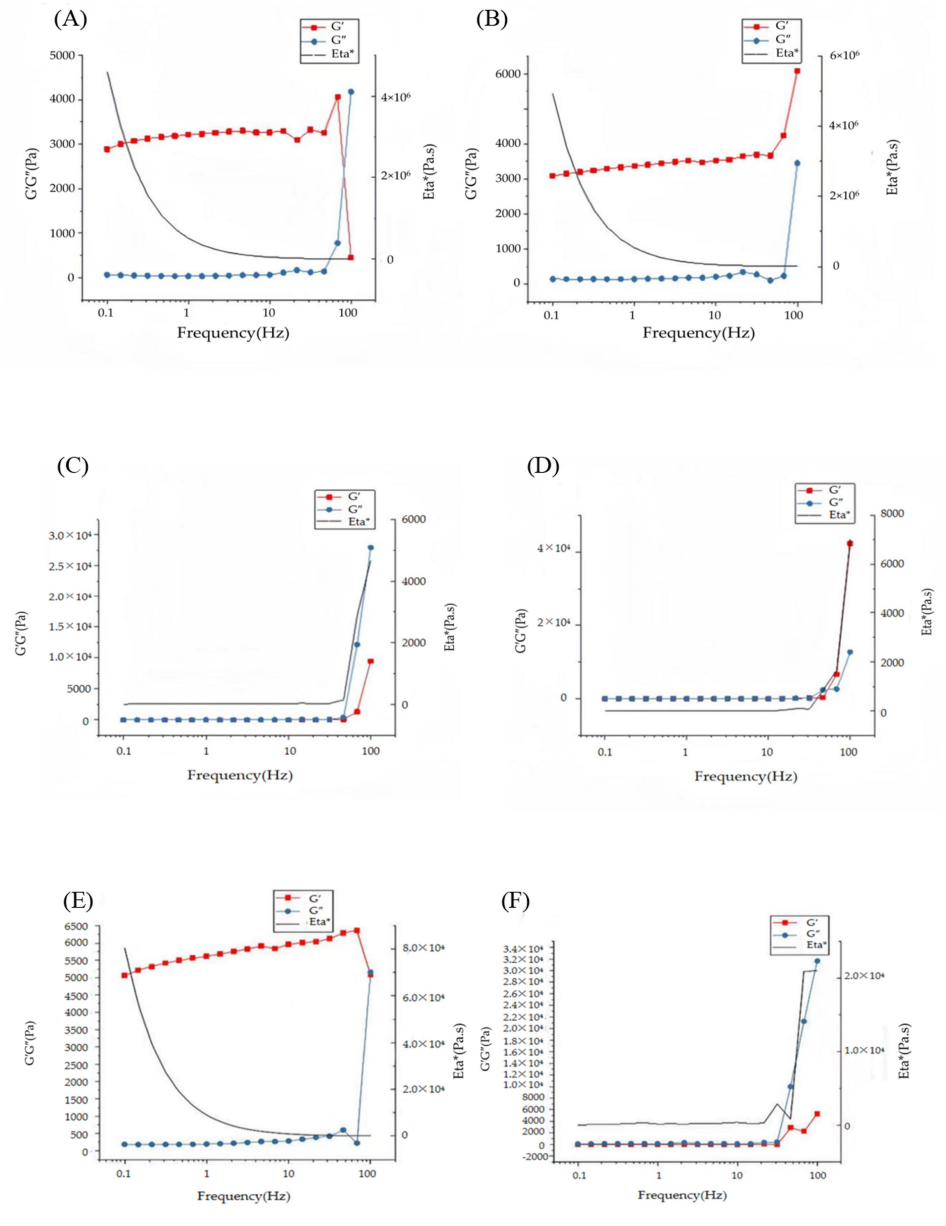
Frequency sweep tests of gel systems. **(A)** 9% (w/v) FG at 10 °C; **(B)** 0.8% (w/v) κ-CG at 10 °C; **(C)** 9% FG at 60 °C; **(D)** 0.8% κ-CG at 70 °C; **(E)** 6.8% FG + 1.2% κ-CG at 10 °C; **(F)** 6.8% FG + 1.2% κ-CG at 70 °C. Mean ± SD, *n* = 3.

Frequency sweep tests revealed distinct viscoelastic differences among FG gel, κ-CG gel, and FG/κ-CG composite gels. At 10 °C, FG gel (9% w/v) displayed elastic-dominated behavior (G′ > G″) at frequencies below 70 Hz (Figure 5A), but shifted to viscous dominance (G″ > G′) at higher frequencies, indicating a weaker structural network which is sensitive to dynamic perturbation. By contrast, κ-CG gel (0.8% w/v) maintained solid-like behavior (G′ consistently greater than G″) across the entire frequency range (0.1–100 Hz; Figure 5B), confirming demonstrating confirming the stability of their rigid double-helix network. The weakening of the FG gel network was more pronounced at elevated temperature (60 °C; Figure 5C), whereas the κ-CG gel network retained its solid-like behavior even at 70 °C (Figure 5D) (28).

Composite 6.8% FG + 1.2% κ-CG gels exhibited improved viscoelasticity compared with FG gel alone. At 10 °C, G′ remained greater than G″ throughout the tested frequency range (Figure 5E), and no crossover was observed. This demonstrated that the dual-network structure effectively reinforced the elastic framework, suppressing the frequency-induced transition to viscous dominance seen in FG gel. At 70 °C (Figure 5F), the composite still maintained G′ > G″, confirming good thermal stability (29). Thus, composites integrated the elasticity of FG with the rigidity of κ-CG, achieving more stable solid-like viscoelasticity across the tested temperatures and frequencies.

The enhanced storage and loss moduli indicate that κ-CG addition reinforces the gel network, improving elasticity and resistance to deformation. Biologically, this mechanical reinforcement provides stability under physiological stresses and maintains gel integrity during digestion or implantation, which is essential for consistent peptide release and tissue interaction.

#### 2.4.2 Temperature-dependent behavior

**Figure 6:**
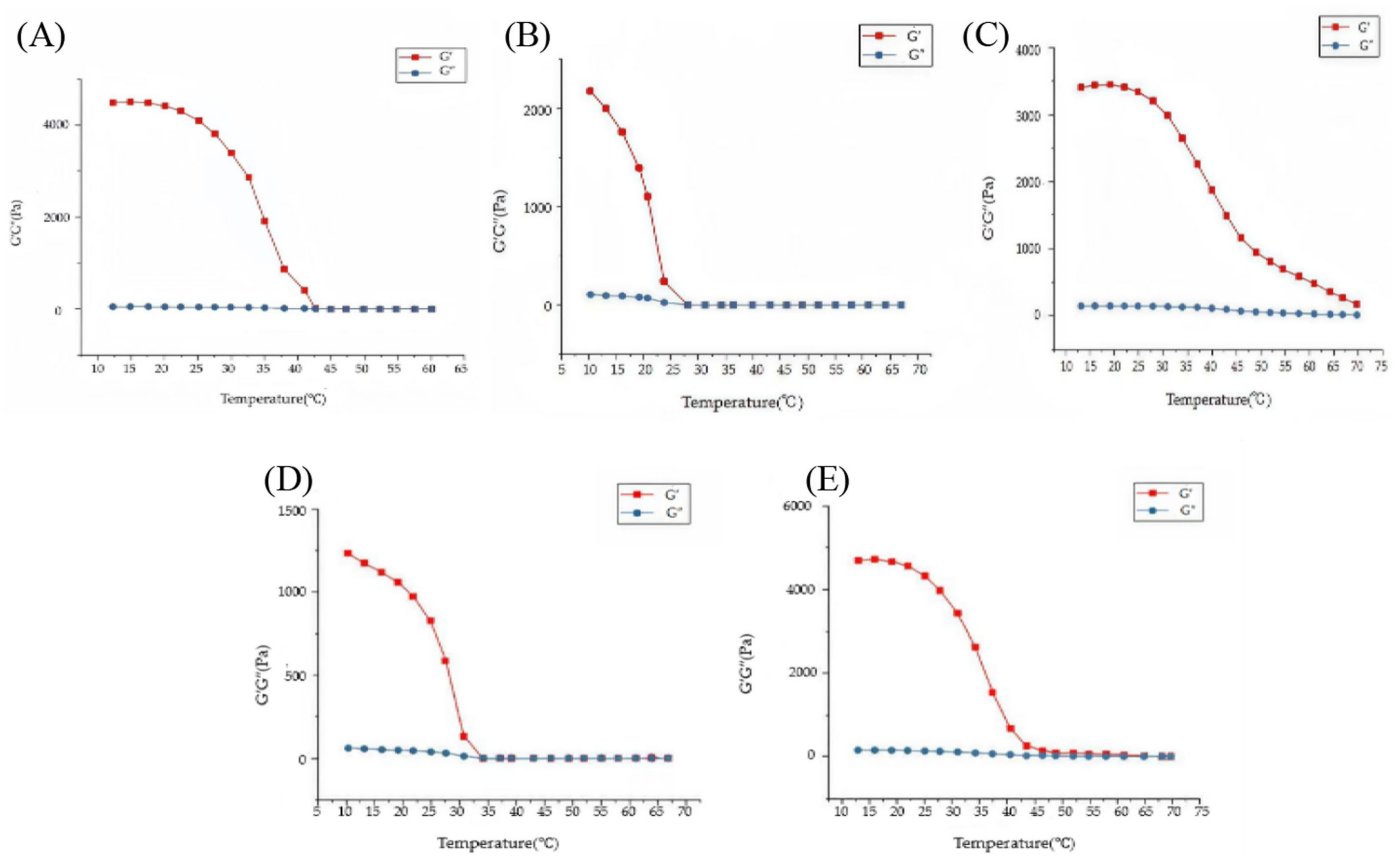
Evolution of viscoelastic moduli (G′ and G″) during temperature ramps for different gel systems. **(A)** 8% (w/v) FG gel during a heating ramp. **(B)** 0.8% (w/v) κ-CG gel during cooling ramps. **(C)** 0.8% (w/v) κ-CG gel during heating ramps. **(D)** 6.8% FG + 1.2% κ-CG composite gel during cooling ramps **(E)** 6.8% FG + 1.2% κ-CG composite gel during heating ramps. Mean ± SD, *n* = 3.

Temperature ramp tests further demonstrated the thermal stability of different gels. FG gel exhibited a sharp melting transition near 40 °C, where G′ rapidly decreased below G″ (Figure 6A), confirming their limited thermal resistance (30). In contrast, κ-CG gel underwent a sol–gel transition upon cooling around 30 °C (Figure 6B) and retained its elastic dominance until melting occurred at 70 °C during heating (Figure 6C), demonstrating superior thermal stability.

Composite 6.8% FG + 1.2% κ-CG gels displayed intermediate but significantly improved thermal behavior. Cooling scans revealed a gelation onset at 35 °C (Figure 6D), slightly higher than FG gel, while heating scans showed a melting point at 65 °C (Figure 6E), approximately 25 °C higher than FG gel and close to κ-CG gel. These results highlighted the synergistic contribution of the κ-CG network in stabilizing the gelatin framework, ensuring that the composite gels not only resist thermal degradation but while also maintain mechanical integrity under physiological and processing conditions.

The improved thermal stability of the FG/κ-CG system arises from dual-network crosslinking between gelatin polypeptide chains and carrageenan sulfate groups. Such stability is crucial for oral or nutritional peptide delivery, as the gel must tolerate temperature and pH fluctuations without premature disintegration. This property also suggests better peptide protection during processing and storage.

The improved mechanical strength and thermal stability observed in the FG/κ-CG gels highlight the advantages of combining protein- and polysaccharide-based networks. To better understand the relevance of these properties, it is helpful to considering how this dual-network structure compares with commonly used peptide carriers is significant. PLGA microspheres, for example, are well known for their high structural rigidity and durability; however, their stiffness and hydrophobic nature often limit their compatibility with soft or highly hydrated biological environments. In contrast, alginate hydrogels provide excellent water retention and biocompatibility but are generally susceptible to mechanical deformation and reduced stability under stress.

Positioned between these two extremes, the FG/κ-CG gel offers a balanced profile. The interpenetrating network created by gelatin’s flexible chains and κ-carrageenan’s ionic interactions grants the material sufficient elasticity while maintaining structural integrity. This combination allows the gel to better accommodate physiological movement and environmental fluctuations without compromising its cohesiveness. Such material behavior is especially relevant for oral or nutritional peptide formulations, where the matrix must remain intact long enough to protect the active component while withstanding temperature variations and hydration changes.

Taken together, these comparisons indicate that the FG/κ-CG system provides a complementary alternative to both rigid synthetic carriers and mechanically weak natural hydrogels. Its balanced mechanical and thermal characteristics support its suitability as a protective matrix for bioactive peptides, laying a strong foundation for the subsequent biological evaluation.

### 2.5 Fluorescent observation and analysis of bone mineralization in zebrafish

The physicochemical improvements described above—namely enhanced hydration, elasticity, and thermal resistance—are expected to translate into better biological performance. To verify this correlation, the FG/κ-CG-SOP gel was evaluated in a glucocorticoid-induced zebrafish osteoporosis model.

**Figure 7:**
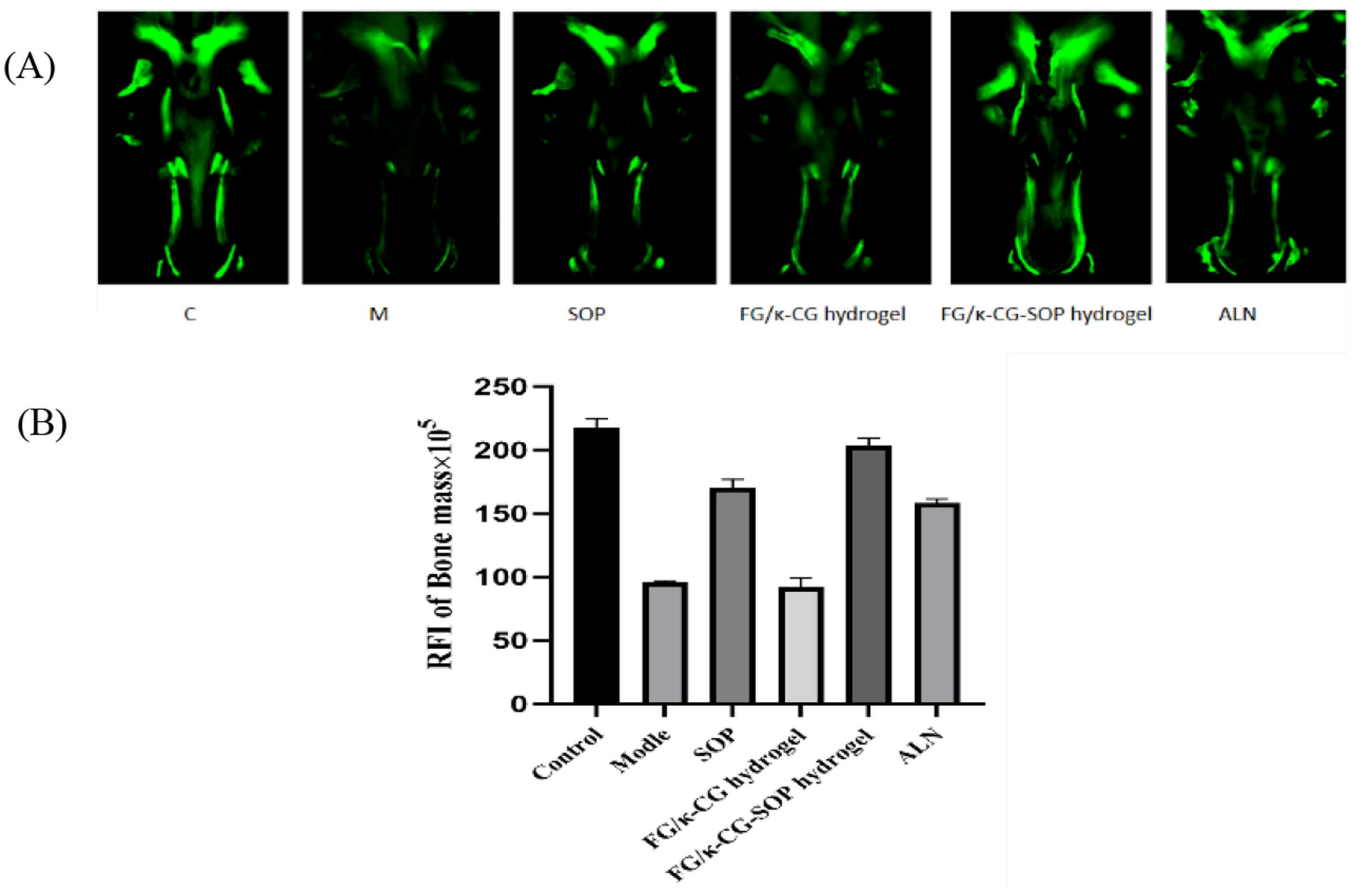
**(A)** Calcein staining and quantitative analysis of bone mineralization in zebrafish. **(B)** Quantitative analysis of relative fluorescence intensity. Mean ± SD, *n* = 11.

Calcein staining of zebrafish cranial bones revealed significant differences in bone mineralization among the treatment groups (Figure 7A). Compared with the blank control group (C), the dexamethasone-induced model group (M) exhibited a marked reduction in fluorescence intensity, confirming the successful establishment of the osteoporosis model. The alendronate sodium group (ALN), used as a positive control, showed strong recovery of fluorescence intensity, approaching normal levels. Treatment with SOP alone partially improved fluorescence signals but remained inferior to the C group, which can be attributed to the susceptibility of SOP to enzymatic degradation in the gastrointestinal tract, thereby limiting its stability and bioavailability *in vivo*. The unloaded dual-network gel group (Group 6.8% FG + 1.2% κ-CG gel) showed fluorescence intensity comparable to the M group, indicating that the gel itself has no therapeutic effect and mainly functions as a carrier to prevent SOP degradation. In contrast, the SOP-loaded dual-network gel group (Group 6.8% FG + 1.2% κ-CG-SOP gel) demonstrated a pronounced increase in fluorescence intensity, comparable to the ALN group, highlighting its superior anti-osteoporotic efficacy through sustained release of SOP.

Quantitative fluorescence analysis further validated these observations (Figure 7B). Compared with the M group, the fluorescence intensity of Group 6.8% FG + 1.2% κ-CG-SOP gel increased by approximately 111.57% and showed no significant difference from the C group. These findings clearly demonstrate that the 6.8% FG + 1.2% κ-CG-SOP gel can effectively restore bone mineralization in osteoporotic zebrafish, achieving therapeutic outcomes comparable to conventional drug treatment.

## 3 Conclusion

In summary, a dual-network hydrogel composed of fish gelatin (FG) and κ-carrageenan (κ-CG) was successfully developed as a stable carrier for the soybean-derived osteogenic peptide (SOP). The integration of protein–polysaccharide networks significantly enhanced the gel’s mechanical strength, hydration stability, and thermal resistance, enabling effective protection and sustained release of the peptide. These physicochemical improvements translated into pronounced osteogenic effects in a zebrafish osteoporosis model, demonstrating the gel’s ability to restore bone mineralization comparable to standard treatment. Overall, the FG/κ-CG-SOP system offers a versatile and biocompatible platform for peptide delivery, holding promise for nutritional supplementation and therapeutic management of osteoporosis.

## 4 Materials and methods

### 4.1 Materials and equipment

Fish gelatin (FG) with model FG-200 (Bloom strength 200 ± 10 g) was a kind gift from Vinh Hoan Collagen Corporation (Vietnam). κ-CG with product code C1013 (food grade, >90% (w/w) κ-type) was purchased from Sigma-Aldrich (USA). SOP (soybean-derived osteogenic peptide, sequence: VVELLKAFEEKF; molecular weight 1,475 Da) was synthesized by solid-phase peptide synthesis (SPPS) and supplied at a purity of ≥95% (Nanjing Peptide Biotechnology Co., Ltd., Nanjing, China). Sodium chloride (NaCl) [analytical grade, ≥99.5% (w/w)] was supplied by Sinopharm Chemical Reagent Co., Ltd. (China). Silicone oil (analytical grade, viscosity 350 cSt) was obtained from Aladdin Biochemical Technology Co., Ltd. (China). All other chemicals are analytical grade and without further purification unless otherwise described.

### 4.2 Preparation and property studies

#### 4.2.1 Preparation of gels

FG gel was prepared by dissolving FG powder in deionized water at concentrations ranging from 6 to 10% (w/v). The mixtures were stirred continuously at 70 °C until complete dissolution, degassed, and subsequently cooled at 4 °C for 8 h to facilitate gelation.

κ-CG gel was obtained by dispersing κ-CG powder in deionized water at concentrations between 0.8 and 1.6% (w/v) under stirring at 70 °C. The solutions were then cooled at 4 °C for 8 h to form gels.

Composite FG/κ-CG gels were fabricated using a solute displacement approach, maintaining a total polymer concentration of 8% (w/v). Specifically, κ-CG was incorporated at concentrations of 0.8, 1.0, 1.2, 1.4, and 1.6% (w/v) by displacing an equivalent mass of FG, resulting in corresponding FG concentrations of 7.2, 7.0, 6.8, 6.6, and 6.4% (w/v). The mixtures were homogenized at 70 °C for 10 min, degassed in a water bath at the same temperature for 30 min, and finally cooled at 4 °C for 8 h to form stable composite gels.

Based on previous studies (13), SOP exhibits optimal osteogenic activity at micromolar concentrations. Therefore, SOP was incorporated into the 6.8% FG + 1.2% κ-CG composite solution at a final concentration of 70 μM prior to the gelation process. The mixture was homogenized at 70 °C for 10 min, degassed for 30 min, and cooled at 4 °C for 8 h to obtain the FG/κ-CG-SOP composite gel.

#### 4.2.2 Sensory evaluation

The morphology, transparency, and color of gels were assessed visually under standardized light conditions. Images were recorded using a digital camera. Colorimetric evaluation was performed using the CIE-Lab* system to quantify changes in brightness and chromaticity. To minimize variability, evaluations were conducted under controlled environmental conditions using the standardized scoring scales outlined in Table 1.

**Table 1:** Determination methods of sensory indicators.

Sensory indicators	Measurement method
Strength	Tear the gelatin by hand; the harder the gelatin is torn, the higher the strength is.
Hardness	Press the gelatin by hand and feel its hardness.
Brittleness	Fold the colloid in half and observe the degree of breakage when it breaks.
Color and transparency	Observe the color and transparency of the gelatin by placing it on white A4 paper.
Syneresis	Use filter paper to absorb the surface water; more water results in a strong syneresis.

Water content $\left(W_{c}\right)$ :

Water content $\left(W_{c}\right)$ was determined by drying the centrifuged samples at 60 °C until a constant weight was achieved. The water content was calculated by:


$$W_{c}(\%)=\frac{m_{1}-m_{d}}{m_{1}}\times 100\%$$

where $m_{d}$ is the dry weight after drying.

#### 4.2.3 Rehydration rate

Dried gelatin samples were immersed in distilled water or physiological saline [0.9% (w/v) NaCl] for 24 h at room temperature to assess their rehydration behavior, reflecting the gel’s ability to absorb and retain moisture. After soaking, surface moisture was carefully removed using filter paper. The rehydration percentage was calculated using:


$$\text{Rehydration Rate}(\%)=\frac{m_{r}-m_{d}}{m_{r}}\times 100\%$$

where $m_{r}$ is the mass after rehydration, and $m_{d}$ is the initial dry mass. This parameter is critical for applications where gels undergo dehydration and subsequent moisture recovery.

#### 4.2.4 Colorimetric analysis

The CIE-Lab* color space was employed for quantitative color evaluation. Measurements of lightness $\left(L^{*}\right)$ , redness-greenness $\left(a^{*}\right)$ , and yellowness-blueness $\left(b^{*}\right)$ values were recorded using a precision colorimeter calibrated before each measurement session. Total color difference (ΔE) relative to reference samples was calculated as:


$$\mathrm{\Delta E}=\sqrt{{\left(L^{*}-L_{0}^{*}\right)}^{2}+(a^{*}-a_{0}^{*})+{\left(b^{*}-b_{0}^{*}\right)}^{2}}$$

where $L_{0}^{*}$ , $a_{0}^{*}$ , $b_{0}^{*}$ represent reference sample values. The parameter ΔE provides a single metric for visual color deviation, essential for product quality control.

#### 4.2.5 Rheological measurements

Dynamic rheological properties of gel samples were characterized using a HAAKE MARS III rheometer equipped with a 35 mm parallel plate geometry. We conducted measurements under controlled strain within the linear viscoelastic region to avoid structural damage. Frequency sweep tests ranged from 0.1 Hz to 100 Hz at a constant temperature of 10 °C to probe viscoelastic behavior.

Temperature sweeps were performed by cooling from 70 °C to 10 °C and heating back from 10 °C to 70 °C at a rate of 2 °C/min to evaluate gelation and melting transitions. The storage modulus G′ (elastic response) and loss modulus G″ (viscous response) were recorded. Gelation point $\left(T_{g}\right)$ and melting point $\left(T_{m}\right)$ were identified as the temperatures at which:


G′=G″

Indicating the transition between liquid-like and solid-like behavior. Apparent viscosity (η′) was also measured as a function of temperature and frequency to characterize the flow resistance of the gels.

### 4.3 *In vivo* experiments

#### 4.3.1 Zebrafish husbandry and maintenance

Wild-type AB-strain zebrafish (*Danio rerio*) were obtained from the China Zebrafish Resource Center (CZRC, Beijing, China). Adults were maintained under standard laboratory conditions (28.5 ± 0.5 °C; 14 h light/10 h dark cycle) in a recirculating aquaculture system. Embryos and larvae were raised in fish water (5.0 mM NaCl, 0.17 mM KCl, 0.33 mM CaCl_2_, and 0.33 mM MgSO_4_) at 28.5 °C. All procedures were approved in accordance with the NIH Guidelines for the Care and Use of Laboratory Animals.

#### 4.3.2 Establishment of GIOP model and experimental grouping

Developing zebrafish embryos at 72 h post-fertilization were collected and randomly divided into six groups (*n* = 11 per group):

- Group C: Blank control group, cultured in standard aquarium water.
- Group M: Model group, cultured in aquarium water containing 1 μM dexamethasone to induce osteoporosis.
- Group SOP: Intervention group, cultured in aquarium water containing 1 μM dexamethasone and 30 μM soybean osteogenic peptide.
- Group 6.8% FG + 1.2% κ-CG gel: Material control group, cultured in an environment containing 1 μM dexamethasone and treated in the presence of unloaded FG/κ-CG dual-network gel (approximately 1 mm^3^).
- Group 6.8% FG + 1.2% κ-CG-SOP gel: Experimental group, cultured in an environment containing 1 μM dexamethasone and co-cultured with SOP-loaded FG/κ-CG-SOP dual-network gel (approximately 1 mm^3^).
- ALN group: Positive control group, cultured in aquarium water containing 1 μM dexamethasone and 0.308 μM sodium alendronate.

All treatments lasted 4 days with daily replacement of fresh medium at corresponding concentrations. At day 7 post-treatment, zebrafish larvae underwent indicator assays.

#### 4.3.3 Skeletal staining

Seven-day-old zebrafish larvae were anesthetized with 0.015% tricaine methanesulfonate (MS-222) and placed in a 2% calcein solution for 6 h under dark conditions. After staining, larvae were rinsed three times with aquarium water to remove surface dye. Larvae were mounted on 3% methylcellulose, and fluorescence images of the head and spinal regions were acquired using a fluorescence microscope under identical exposure parameters.

### 4.4 Statistical analysis

Data processing, statistical analysis, and graphing were performed using Origin 8.0 and GraphPad Prism 9.5. Quantitative data are expressed as mean ± SD. Differences between groups were analyzed using ANOVA. A *p*-value < 0.05 was considered statistically significant, while ns indicated no significant difference.

## Data Availability

The original contributions presented in the study are included in the article/supplementary material, further inquiries can be directed to the corresponding author.
